# A phylogenetically conserved APETALA2/ETHYLENE RESPONSE FACTOR, ERF12, regulates *Arabidopsis* floral development

**DOI:** 10.1007/s11103-019-00936-5

**Published:** 2019-12-05

**Authors:** J. W. Chandler, W. Werr

**Affiliations:** Developmental Biology, Institute of Zoology, Cologne Biocenter, University of Cologne, Zuelpicher Straße 47b, 50674 Cologne, Germany

**Keywords:** Angiosperm evolution, APETALA2, ETHYLENE RESPONSE FACTOR 12, Floral transition, Meristem identity, MULTIFLORET SPIKELET1, Phyllotaxy, Supernumerary sepals

## Abstract

**Key message:**

*Arabidopsis ETHYLENE RESPONSE FACTOR12* (*ERF12*), the rice *MULTIFLORET SPIKELET1* orthologue pleiotropically affects meristem identity, floral phyllotaxy and organ initiation and is conserved among angiosperms.

**Abstract:**

Reproductive development necessitates the coordinated regulation of meristem identity and maturation and lateral organ initiation via positive and negative regulators and network integrators. We have identified *ETHYLENE RESPONSE FACTOR12* (*ERF12*) as the *Arabidopsis* orthologue of *MULTIFLORET SPIKELET1* (*MFS1*) in rice. Loss of *ERF12* function pleiotropically affects reproductive development, including defective floral phyllotaxy and increased floral organ merosity, especially supernumerary sepals, at incomplete penetrance in the first-formed flowers. Wildtype floral organ number in early formed flowers is labile, demonstrating that floral meristem maturation involves the stabilisation of positional information for organogenesis, as well as appropriate identity. A subset of *erf12* phenotypes partly defines a narrow developmental time window, suggesting that *ERF12* functions heterochronically to fine-tune stochastic variation in wild type floral number and similar to *MFS1*, promotes meristem identity. *ERF12* expression encircles incipient floral primordia in the inflorescence meristem periphery and is strong throughout the floral meristem and intersepal regions. *ERF12* is a putative transcriptional repressor and genetically opposes the function of its relatives *DORNRÖSCHEN*, *DORNRÖSCHEN*-*LIKE* and *PUCHI* and converges with the *APETALA2* pathway. Phylogenetic analysis suggests that *ERF12* is conserved among all eudicots and appeared in angiosperm evolution concomitant with the generation of floral diversity.

**Electronic supplementary material:**

The online version of this article (10.1007/s11103-019-00936-5) contains supplementary material, which is available to authorized users.

## Introduction

Plant reproductive development involves successive phase changes, whereby vegetative apical meristems that initiate leaf primordia at their periphery become inflorescence meristems (IMs) and initiate floral meristems, which in turn, generate floral organs. The gene regulatory networks that regulate these meristem identities have been elucidated in detail (Ó’Maoiléidigh et al. 2014), together with those involved in the floral transition in response to seasonal cues, for representative dicot and monocot species (Shrestha et al. 2014). Reproductive phase change also depends on endogenous age-related competencies that integrate into these genetic networks via miRNAs (Teotia and Tang 2015). Flower development thus proceeds from the complex coordinated specification of meristem identity, and floral organ initiation and identity, via positive and negative regulators and network integrators.

Instead of leaf initiation at the shoot apical meristem switching to flower production within a single plastochron, the acquisition and maintenance of floral meristem (FM) identity are not robust and can revert to IM identity in several species in response to environmental conditions (McCullough et al. 2010; Tooke et al. 2005). Furthermore, floral organs do not initiate simultaneously within the FM in concentric whorls: instead, outer whorl organs initiate sequentially along an abaxial/adaxial axis, with a stereotypy that can be disrupted by bract founder cell recruitment, or gene mutations that lead to pentameric asymmetry (Chandler and Werr 2014). The floral transition and floral organ initiation therefore represent the outread of multiple components and incremental signals and consist of several distinct phases.

Threshold expression models have been invoked to explain the function of some master regulators of the floral transition, such as *LEAFY* (*LFY*) (Blázquez et al. 1997), and the control of *APETALA2* (*AP2*) by miRNAs (Chen 2004). Many master regulators such as *LFY*, function pleiotropically in both reproductive timing and floral identity (Schultz and Haughn 1991; Blázquez et al. 1997), and in addition to regulating the identity of the outer two floral organ whorls, *APETALA2* (*AP2*) represses the floral transition (Yant et al. 2010). *Arabidopsis* AP2 is the founding member of the large AP2 superfamily of transcription factors, which has been subdivided according to the presence of one or two AP2 domains (Nakano et al. 2006). AINTEGUMENTA (ANT) subclass AP2 members contain two AP2 domains and play developmental roles in *Arabidopsis* (Horstman et al. 2014) and also mediate resistance to salt stress (Meng et al. 2015). Most single AP2-domain proteins belong to the ethylene response element (ERF), also known as ethylene response element binding protein (EREBP) subgroup (Nakano et al. 2006). Although ERF proteins mediate biotic and abiotic stress responses in many taxa (Dey and Corina Vlot 2015: Müller and Munné-Bosch 2015), the group VIIIb members DORNRÖSCHEN (DRN), DORNRÖSCHEN-LIKE (DRNL), LEAFY PETIOLE and PUCHI also regulate development in diverse plant species (Chandler 2018). Given the high sequence conservation of the AP2 domain among AP2 family members, genetic redundancy is a common feature. For example, *ANT*-*like* genes provide distinct individual contributions towards flower development, but function redundantly with *ANT* (Krizek 2015), and *DRN*, *DRNL* and *PUCHI* redundantly regulate organ number and floral meristem identity (Chandler and Werr 2017). The subtle nature of individual mutant phenotypes and the ubiquity of higher orders of genetic redundancy, suggest that the full repertoire of developmental functions of AP2 family members might not yet have been elucidated.

The comparative analysis of AP2 transcription factor functions can inform their evolution and establish plesiomorphic functions. For example, AP2/ERF members in monocots have revealed evolutionary divergence and functional conservation. In rice, the AP2-type genes *SUPERNUMERARY BRACT* and *OSINDETERMINATE SPIKELET1* synergistically determine inflorescence architecture and floral meristem identity (Lee and An 2012). Similarly, *INDETERMINATE SPIKELET* (*IDS*) and *SISTER OF INDETERMINATE SPIKELET1* (*SID1*) play multiple roles in regulating maize inflorescence architecture and although they share some gene targets and functions with *Arabidopsis* AP2, have also adopted novel functions (Chuck et al. 2008). Monocot counterparts of the clade VIIIb ERF subfamily in *Arabidopsis* have also neofunctionalised or retained ancestral functions: in contrast to a mild mutant phenotype in *Arabidopsis*, mutations in the *PUCHI* orthologues *BRANCHED SILKLESS* in maize (Colombo et al. 1998) and *FRIZZY PANICLE* in rice cause much more severe phenotypes (Komatsu et al. 2003). Inter-taxa studies are important not least because *Arabidopsis* inflorescence development is not representative of the whole plant kingdom, due to the different inflorescence structure of monocots versus dicots, including branching, and multiple meristem identities and transitions (Whipple 2017). Furthermore, grasses such as rice represent a specialised and derived lineage of monocots (Kellogg 2001).

Here, we describe the function of *ERF12* in *Arabidopsis*, the orthologue of rice *MULTIFLORET SPIKELET1*, which regulates floral organ identity and number, and the timing of spikelet initiation (Ren et al. 2013). Loss of *ERF12* function affects pleiotropic aspects of floral development in long days, including floral meristem phyllotaxy, organ merosity in the first-formed flowers, notably in sepal number, and delays the floral transition in long and short days. These phenotypes, which are partly restricted to a narrow developmental time window, suggest that *ERF12* possesses a heterochronic function, which regulates the timing of FM specification and fine-tunes the inherent wild type stochastic variation in floral organ merosity in first-formed flowers. Phylogenetic analysis of the ERF12 AP2 domain suggests that ERF12 represents a highly conserved angiosperm-specific innovation.

## Materials and methods

### Plant material and growth conditions

The mutant line SAIL_873_D11 (N877578) contains a T-DNA insertion in the open reading frame of the *ERF12* gene (*At1G28360*) after nucleotide 427 from the ATG start. For *erf9*, the line SALK_043407 contains a T-DNA insertion after nucleotide 314 from the ATG start in the *ERF9* (*At5G44210*) open reading frame. Both *erf12* and *erf9* alleles were obtained from NASC. Homozygosity was confirmed by genotyping using primers ERF12genoF/ERF12genoR to genomic DNA spanning the open reading frame, or ERF12F/LB3 for the presence of the insertion for *erf12*, or using primers ERF9F/ERF9R or Lba1/ERF9R for *erf9*. The mutant lines *drn*-*1*, *drnl*-*1* and *puchi* and their genotyping have been described previously (Chandler et al. 2007; Chandler and Werr 2017). The *ap2*-*7* allele from NASC (N6241) is in Col-0 background. Plants were grown on soil in the greenhouse in long-day conditions (16 h light, 8 h dark) or in a controlled environment cabinet at 100 µM m^−2^ light intensity under short days (8 h dark, 16 h light).

### Constructs for expression analysis and complementation

For expression analysis, 2356 bp *ERF12* upstream promoter sequence from the stop codon of the upstream gene to the start codon of the *ERF12* open reading frame (*AT1G28360*) was amplified from *Arabidopsis* genomic DNA extracted from wild-type plants using the NucleoSpin® Plant II kit (Macherey–Nagel) and primers promERF12F and promERF12R. The resulting fragment was cloned into the TOPO™ TA Cloning™ vector (ThermoFisher Scientific). The primers included flanking *Asc*I (5′) and *Xma*I (3′) sites, the latter allowing the introduction the coding region of the green fluorescent protein targeted to the endoplasmic reticulum (*erGFP*) sequence that terminates in the cauliflower mosaic virus 35S 3′UTR/polyadenylation signal in front of a second *Asc*I restriction site (Comelli et al. 2016). The orientation of *erGFP* relative to the *ERF12* promoter was confirmed by sequencing, to produce *pERF12::erGFP*. Two genomic regions containing the *ERF12* open reading frame were used for complementation analysis. The shorter version (*gERF12Up*; 3527 bp) was amplified using primers promERF12F and gERF12UpR, to include the whole *ERF12* upstream promoter region from the stop codon of the upstream gene, the *ERF12* open reading frame and the 3′ untranslated region. Both primers included flanking *Asc*I sites. A longer genomic region (*gERF12UpDown*; 6773 bp) was constructed, consisting of the whole genomic region, between the open reading frames of the up- and downstream genes, including the *ERF12*-coding region. This construct was made in two steps, by firstly reamplifying *gERF12Up* using primers promERF12F and gERF12UpRnew, the latter containing an *Xma*I site and cloning the product into TOPO™ TA Cloning™ vector (ThermoFisher Scientific) and then introducing a PCR fragment amplified using primers gERF12DSF and gERF12DSR at the 3′ end of *gERF12Up* via the flanking *Xma*I sites and confirming the correct orientation in relation to the *gERF12Up* fragment, to create *gERF12UpDown*. The *pERF12::erGFP* expression or complementation cassettes were transferred into the binary pGPTV-Asc-Bar (*erGFP* expression construct) or pGPTV-Asc-Kan (complementation constructs) vectors (Überlacker and Werr 1996) via *Asc*I and were introduced into *Agrobacterium tumefaciens* GV3101V. Mutant *erf12* plants (for complementation) or Col-0 wild type (*pERF12::erGFP*) were transformed via floral dipping (Clough and Bent 1998). For expression analysis, transgenic plants for *pERF12::erGFP* were selected with BASTA and at least eight independent T_2_ lines were analysed. For complementation, T_1_ seeds were surface-sterilised with bleach (Lindsey et al. 2017). Plants were selected on sterile half-strength Murashige and Skoog medium for resistance to kanamycin (50 µg mL^−1^) and were genotyped to confirm the presence of the appropriate transgene using pGPTV-specific pGPTVF/pGPTVR and gene-specific primers ERF12Fgeno/ERF12Rgeno. T_2_ populations of several independent transgenic lines were analysed for complementation.

### Confocal imaging

Imaging of *pERF12::erGFP* expression was performed with a Zeiss LSM 700 confocal laser scanning microscope (CLSM). GFP was excited at 488 nm and emission was analysed between 502 and 525 nm. Photoshop CS2 software (Adobe) was used to process the CLSM images and Imaris software (Bitplane, Zürich, Switzerland) converted Z-stacks into 3D images.

### Phenotypic analysis

Organ numbers were counted for each whorl of the first five-formed flowers for 100 plants. Statistical analyses of organ numbers were performed using *t* tests.

### Phylogenetic analysis and phylogenetic shadowing

Full-length protein sequences were obtained from GenBank (http://www.ncbi.nlm.nih.gov/genbank/) or the PLAZA databases (https://bioinformatics.psb.ugent.be/plaza/) using ERF12 or MFS1 sequences as a query. A phylogenetic tree was compiled using the phylogeny.fr platform (Dereeper et al. 2008); protein sequences were aligned using the MUSCLE program and tree construction employed the maximum likelihood method (PhyML programme) and the Bootstrap procedure (100 replicates). The phylogenetic tree was rendered with TreeDyn software and Bootstrap support values are indicated next to the branches. For phylogenetic shadowing, *ERF12* genomic regions of Brassicacea species (*Arabidopsis thaliana, Arabidopsis lyrata*, *Boechera stricta*, *Camelina sativa*, *Eutrema salsugineum* and *Capsella rubella*) were obtained from http://phytozome.jgi.doe.gov/pz/portal.html or http://www.ncbi.nlm.nih.gov/. Sequences were analysed with online mVista tool with LAGAN alignment (http://genome.lbl.gov/vista/index.shtml).

## Results

### ERF12 and MFS1 represent an invention that postdates basal angiosperms

**Fig. 1:**
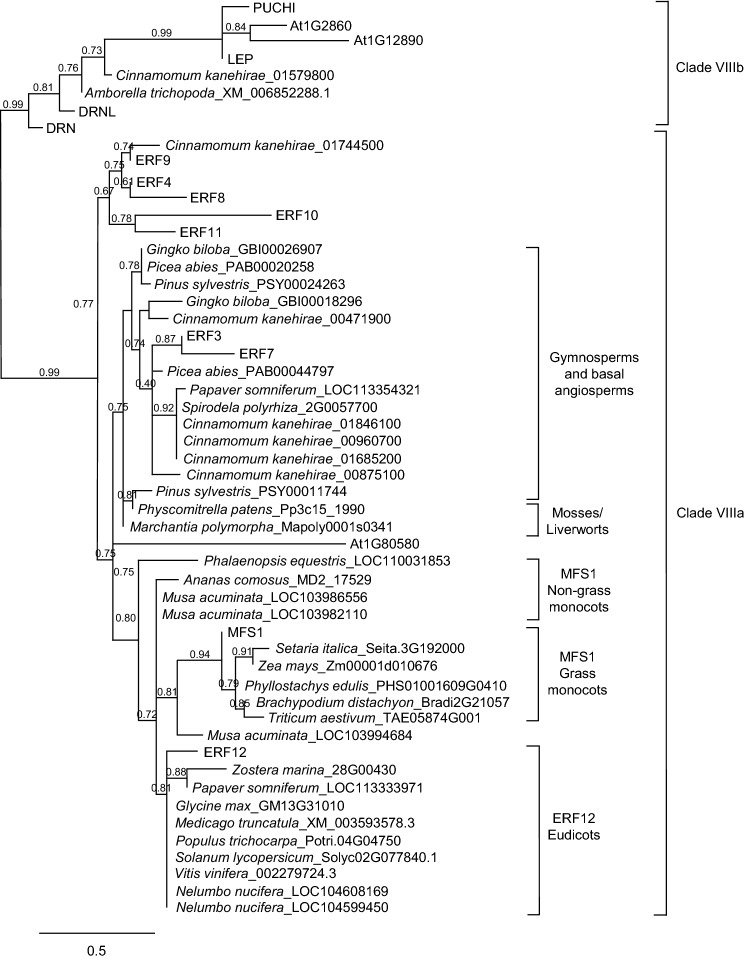
Phylogenetic relationship between *Arabidopsis* class VIIIa and VIIIb ERF/AP2 transcription factors and ERF12/MFS1 homologues from diverse plant taxa. The tree was compiled using the maximum likelihood method (PhyML programme), via the phylogeny.fr platform (Dereeper et al. 2008)

**Fig. 2:**
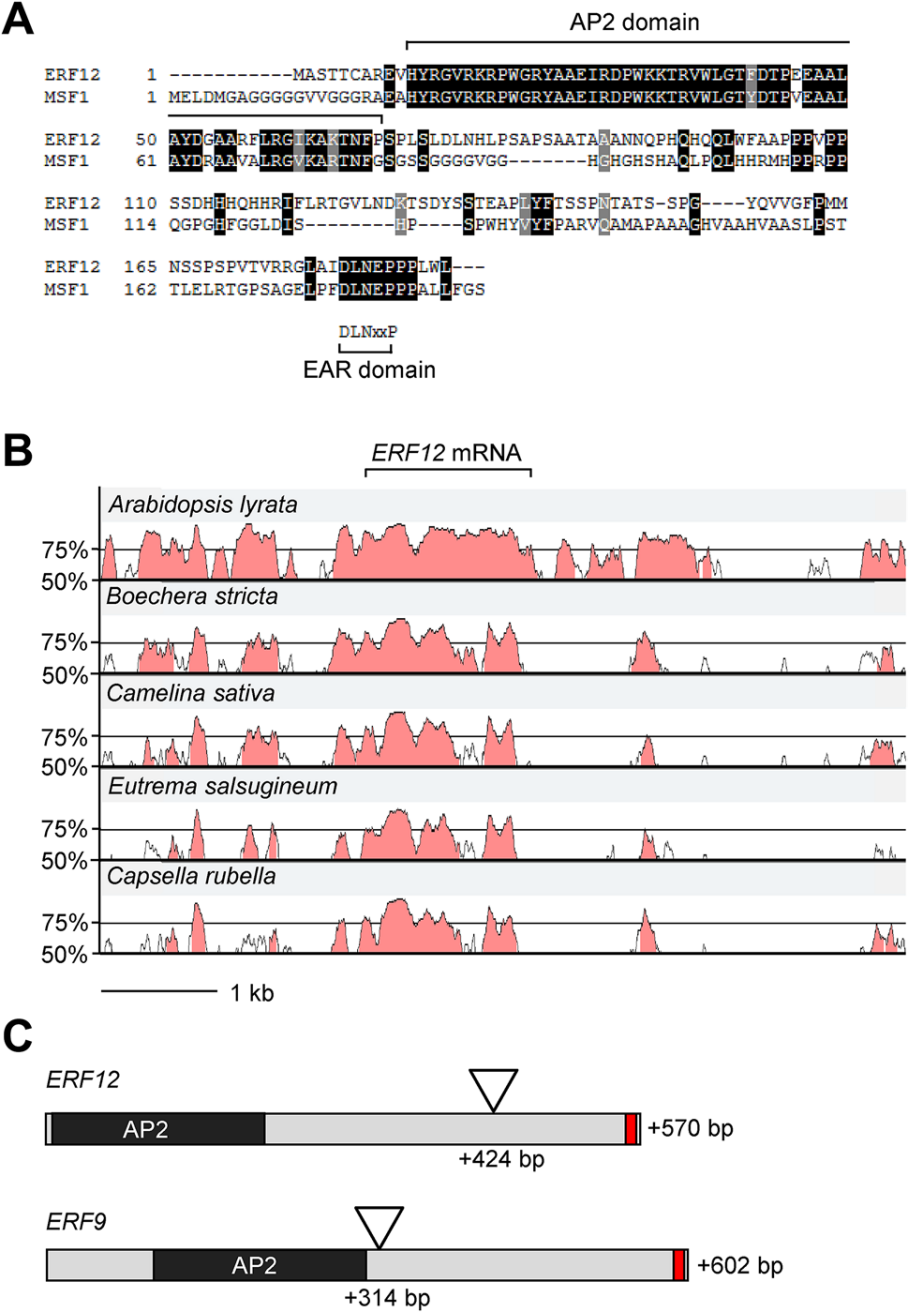
Position of *erf12* mutation and conservation of ERF12 protein domains and regulatory regions. Amino acid alignment of MFS1 from rice and Arabidopsis ERF12 (**a**) showing high conservation in the AP2 and EAR domains. VISTA blots of the *ERF12* genomic locus, including the up- and downstream flanking genes compared between *Arabidopsis thaliana* and a range other Brassicaceae species (*Arabidopsis lyrata*, *Boechera stricta*, *Camelina sativa*, *Eutrema salsugineum* and *Capsella rubella*) (**b**). Schematic representation of the *ERF9* and *ERF12* open reading frames (**c**) showing the position of the T-DNA insertions and the encoded AP2 domain and EAR domain (red block)

A blast search using the MFS1 amino acid sequence revealed *Arabidopsis* ERF12 to be a putative orthologue. ERF12 belongs to sub-clade VIIIa of AP2/ERF proteins (Nakano et al. 2006), which contains eight members (ERF3, ERF4, ERF7–12). To address the evolutionary sequence conservation between rice MFS1 and *Arabidopsis* ERF12, a phylogenetic analysis of the closest available full-length-protein sequences to ERF12 and MFS1 was performed from a range of representative species from different extant phylogenetic groups. All members of clade VIIIa and VIIIb were included in the comparison, together with the closest one, or occasionally two sequences from blast searches made with ERF12 and MFS1 against genomes of mosses and liverworts (*Marchantia polymorpha*, *Physcomitrella patens*), basal angiosperms (*Amborella trichopoda* and the mangoliid *Cinnamomum kanehirae*), gymnosperms (*Gingko* biloba, *Picea abies*, *Pinus sylvestris*), grass monocots (*Brachypodium distachyon*, *Phyllostachys edulis*, *Setaria italica*, *Triticum aestivum*, *Zea mays*), non-grass monocots (*Ananas comosum*, *Musa acuminata*, *Phalaenopsis equestris*, *Spirodela polyrhiza*, *Zostera marina*), basal eudicots (*Nelumbo nucifera*, *Papaver somniferum*), core eudicots (*Glycine max*, *Medicago truncatula*, *Populus trichocarpa*, *Solanum lycopersicum* and *Vitis vinifera*). These sequences formed monocot and eudicot ERF12 subclades, but the closest gymnosperm, moss and liverwort ERF12 and MFS1 homologues clustered more closely with *Arabidopsis* subclades ERF3 and ERF7 (Fig. 1). Furthermore, the closest ERF12/MFS1 homologue in the angiosperm sister taxon *Amborella trichopoda* aligned most closely with DRNL (Fig. 1), suggesting that ERF12 proteins represent an invention subsequent to the divergence of gymnosperms and basal angiosperms. The AP2 domain is highly conserved between ERF12 and MFS1 and both proteins share a C-terminal EAR-like domain (Fig. 2a). To assess the conservation of *ERF12* regulatory sequences, phylogenetic shadowing of the genomic *ERF12* locus among several Brassicaceae species revealed that upstream *ERF12* promoter sequences are more highly conserved than downstream regulatory sequences (Fig. 2b).

### *ERF12* promotes the floral transition in long and short days

**Fig. 3:**
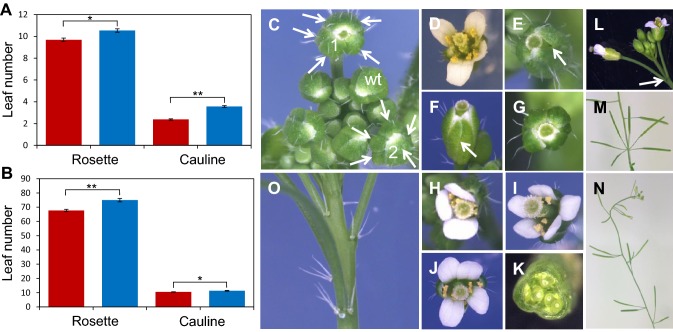
*erf12* mutant phenotype. Flowering time as indicated by rosette and cauline and leaf number for wild type (red bars) and *erf12* plants (blue bars) in long day (**a**) and short day (**b**) photoperiods; **p ≤ 0.001; **p ≤ 0.0001. Sepal phenotypes of *erf12* mutants: top-view of young inflorescences (**c**) showing supernumerary sepals (all sepals marked by arrows) in the first two flowers (marked 1 and 2). A wild-type bud with four sepals is marked “wt”. A wild-type flower with four petals and six stamens clearly visible (**d**). Single buds showing fused supernumerary abaxial sepals (**e**; fusion marked with arrows) or supernumerary sepals at various positions (**f**, **g**). *erf12* flowers with three (**h**) or five (**i**) petals; seven stamens (**j**) and three fused carpels (**k**). Floral phyllotaxy defects in inflorescences of *erf12* mutants: the first two flowers occupying the same stem node separated by an acute angle (**l**), or clustering of flowers due to aberrant phyllotaxy and internode elongation (**m**, **n**). Trichomes at the position of the cryptic bract, subtending floral pedicels of *drn drnl puchi* mutants (**o**)

To characterise the function of *ERF12*, an *erf12* mutant was analysed that contained a T-DNA insertion within the *ERF12* open reading frame (Fig. 2a). As a proxy for the timing of the floral transition, the number of rosette and cauline leaves were all significantly higher (0.001 > *p* < 0.0001) for *erf12* than wild type in both long and short days (Fig. 3a, b).

### Loss of *ERF12* function preferentially affects sepal and stamen number in long days

**Fig. 4:**
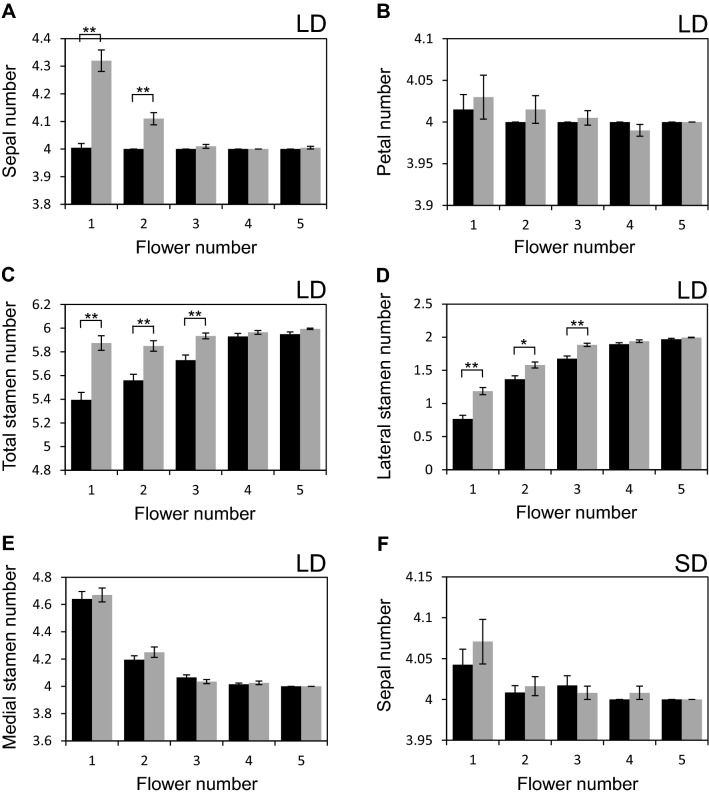
*S*epal, petal and stamen number is labile in earliest-formed wild type and *erf12* flowers. Frequency of sepals (**a**); petals (**b**); total stamens (**c**); lateral stamens (**d**); medial stamens (**e**) in the first five flowers of wild type and *erf12* mutants grown in long days. N = 200; *p < 0.05; **p < 0.0001. Frequency of sepals in the first five flowers of wild type and *erf12* mutants grown in short days (F) (n = 117 for wild type; 123 for *erf12*). Black and grey columns represent wild type and *erf12*, respectively. *LD* long days, *SD* short days. Flower number 1 represents the first-formed flower

Petal and stamen frequency in *erf12* mutants grown in long days showed the same qualitative trend across the first few flowers in the inflorescence as wild type, but differed quantitatively. The most pronounced *erf12* floral phenotype was significantly more sepals in the first two-formed flowers than wild type (Figs. 3c, e–g, 4a) at a penetrance that was highest in first-formed flowers (24%) and decreased to 10% in flower 2 and 1% in flower 3, but ectopic sepals were observed up to flower 6. Sepals were also occasionally fused (in 11% of first-formed flowers; Fig. 3d, e). Mean petal number in the first few *erf12* flowers was slightly but not significantly greater than in wild type (Fig. 4b), but more *erf12* flowers had either three or five petals than wild type (Fig. 3h, 1; data not shown), which usually had four (Fig. 3d). *erf12* flowers had significantly more stamens than wild type (up to nine) in the first three flowers (Figs. 3j, 4c), preferentially due to significantly more lateral stamens (Fig. 4d); the number of medial stamens in flowers 1 to 5 did not differ between wild type and *erf12* plants (Fig. 4e), but reached seven in both genotypes. Three fused carpels were observed among the first five *erf12* flowers at a very low frequency (Fig. 3k; 1/200 plants). Thus, early *erf12* flowers possessed supernumerary organs in all four floral whorls.

Notably, floral organ number in earliest-formed wild-type flowers was non-stereotypic in long-day conditions and was not robust across the inflorescence. No significant variation in sepal number was observed in the earliest-formed flowers (Fig. 4a), but first-formed flowers had more petals than subsequent flowers (Fig. 4b). However, a significant increase total stamen number was observed, from 5.40 in the first-formed flower to 5.95 in flower 5 (Fig. 4c), which resulted from opposing trends in the number of lateral and medial stamens, with the former increasing to a greater extent than the decrease in the number of medial stamens (Fig. 4d, e). In addition to mean stamen number, approximately half (46.5%) of wild type flowers possessed more than six and up to nine stamens. These supernumerary stamens were always medial, on either or both ab- or adaxial sides of the flower.

Primary or secondary *erf12* inflorescences showed phyllotactic defects at almost complete penetrance (98%; N = 99 inflorescences), resulting from an aberrant divergence angle of siliques on the stem, or inhibited internode elongation, to give clustered siliques (Fig. 3l–n). These defects were maintained throughout inflorescence development and were not lost in later-initiated flowers (visible in Fig. 3n). In controlled short-day growth conditions, the number of wild type and *erf12* floral organs did not differ significantly (Fig. 4f for sepals).

### *ERF12* is expressed dynamically in the IM and FM

**Fig. 5:**
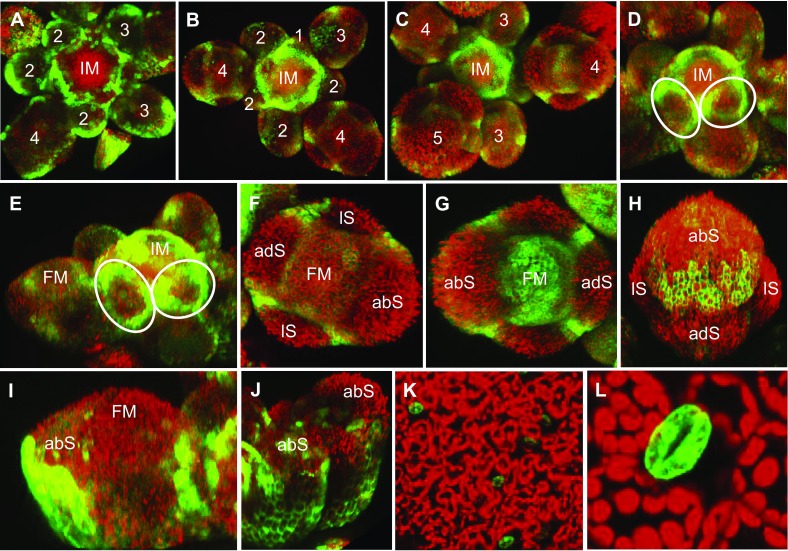
*ERF12* is dynamically expressed in developing flowers and in guard cells. Expression of *pERF12::erGFP* in top views of inflorescence apices from independent transgenic lines showing flowers in different stages of development (**a**–**c**); oblique views of inflorescence apices showing rings of *pERF12::erGFP* expression surrounding the emerging floral primordia (marked by oblongs) (**d**, **e**); top views of early (**f**) and late stage 4 (**g**) and stage 6 (**h**) floral buds; saggital view of stage 4 floral buds (**i**, **j**); epidermis of a cotyledon (**k**); a leaf (**l**). *IM* inflorescence meristem, *FM* floral meristem, *abS* abaxial sepal, *adS* adaxial sepal, *lS* lateral sepal; numbers represent floral stages according to Smyth et al. (1990)

*ERF12::erGFP* was dynamically expressed in the IM and FM during inflorescence development (Fig. 5a–c). Strongest expression was at the IM periphery at the border between newly initiated FMs from about P1 stage onwards, around the basal circumference of the floral primordium, as soon as the buttresses physically extended from the IM (Fig. 5d, e). In buds from about stage 3 onwards, foci of *ERF12::GFP* expression marked the intersepal regions in floral buds and diffuse expression was present internally to the sepals throughout the FM (Fig. 5f, g) and in the tips of sepals as they overgrew the FM (Fig. 5h). Strong expression was also observed at the abaxial underside flank of the developing bud (Fig. 5i, j) and in guard cells of cotyledons and leaves (Fig. 5k, l).

### Complementation of the *erf12* mutant

**Fig. 6:**
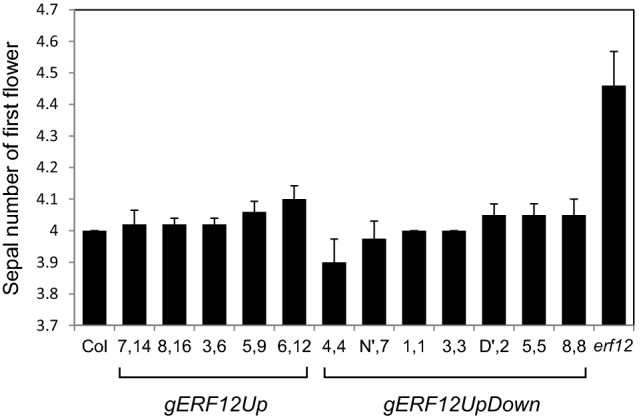
Complementation of the *erf12* mutant. Sepal number in the first-formed flower of multiple independent *erf12* transgenic lines complemented with the *ERF12* locus containing upstream (*gERF12Up*) or up- and downstream regulatory sequences (*gERF12UpDown*)

Sepal number in the first-formed flower was used to demonstrate complementation by genomic *ERF12* sequences containing the *ERF12* open reading frame and either the upstream genomic region (*gERF12Up*) or all upstream and downstream sequences (*gERF12UpDown*). Five and seven independent transgenic lines containing *gERF12Up* or *gERF12UpDown*, respectively, all showed significantly statistically fewer sepals in the first-formed flower than *erf12* mutants (Fig. 6), mostly to the wild-type number of four. The late flowering *erf12* phenotype was not complemented (data not shown).

### Genetic interactions between *erf12*, *drn*, *drnl* and *puchi*

**Fig. 7:**
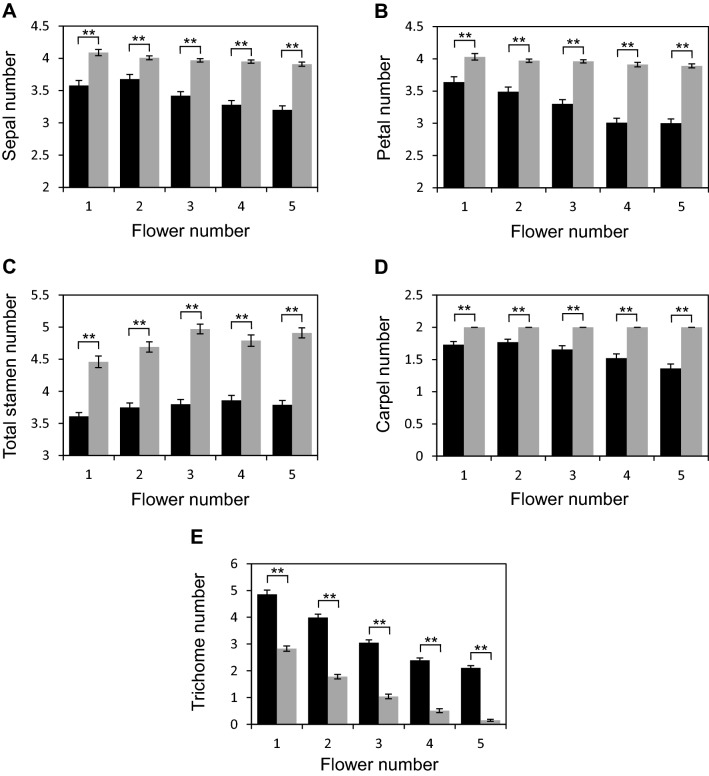
*ERF12* interacts genetically with related AP2/ERF genes *DORNRÖSCHEN* (*DRN*), *DORNRÖSCHEN*-*LIKE* (*DRNL*) and *PUCHI*. Frequency of sepals (**a**); petals (**b**); total stamens (**c**); carpels (**d**): trichomes subtending the pedicels (**e**) in *drn drnl puchi* and *erf12 drn drnl puchi* mutants. Black and grey columns represent *drn drnl puchi* and *erf12 drn drnl puchi* mutants, respectively. Flower number 1 represents the first-formed flower. N = 200; ** *p* ≤ 0.0001

The related AP2/ERF genes *DRN*, *DRNL* and *PUCHI* redundantly regulate floral organ number in all whorls and *drn drnl puchi* mutants display trichomes at the base of floral pedicels at the position of the cryptic bract (Fig. 3o). We therefore investigated potential genetic interactions between these genes and *ERF12* by creating a quadruple *drn drnl puchi erf12* mutant, which was not trivial, due to linkage between *DRN*, *DRNL* and *ERF12* loci on chromosome 1. Similar to wild type, *drn drnl puchi* triple mutant flowers showed a gradient of organ numbers throughout inflorescence development, with a decrease in the number of trichomes subtending the pedicels, fewer sepals and petals and more stamens, from the first to the fifth flower (Fig. 7a–e). The *erf12* phenotype was not additive, synergistic or epistatic when combined with *drn drnl puchi*. Instead, the frequency of trichomes subtending the first-formed flowers of *drn drnl puchi erf12* quadruple mutants was attenuated (Fig. 7e), and the number of floral organs in all whorls was significantly higher than in *drn drnl puchi* triple mutants, although remained lower than wild type (Fig. 7a–d), except for the frequency of carpels, which was restored to the wild type number of two (Fig. 7d). Sepal number in the first few *drn drnl puchi erf12* flowers was significantly lower than in single *erf12* mutants. The penetrance of cotyledon defects in *drn drnl puchi* mutants was 46.16% (N = 2110), and was attenuated in *erf12 drn drnl puchi* mutants to 30.94% (N = 1939).

### *erf12* enhances the *ap2* mutant phentoype

**Fig. 8:**
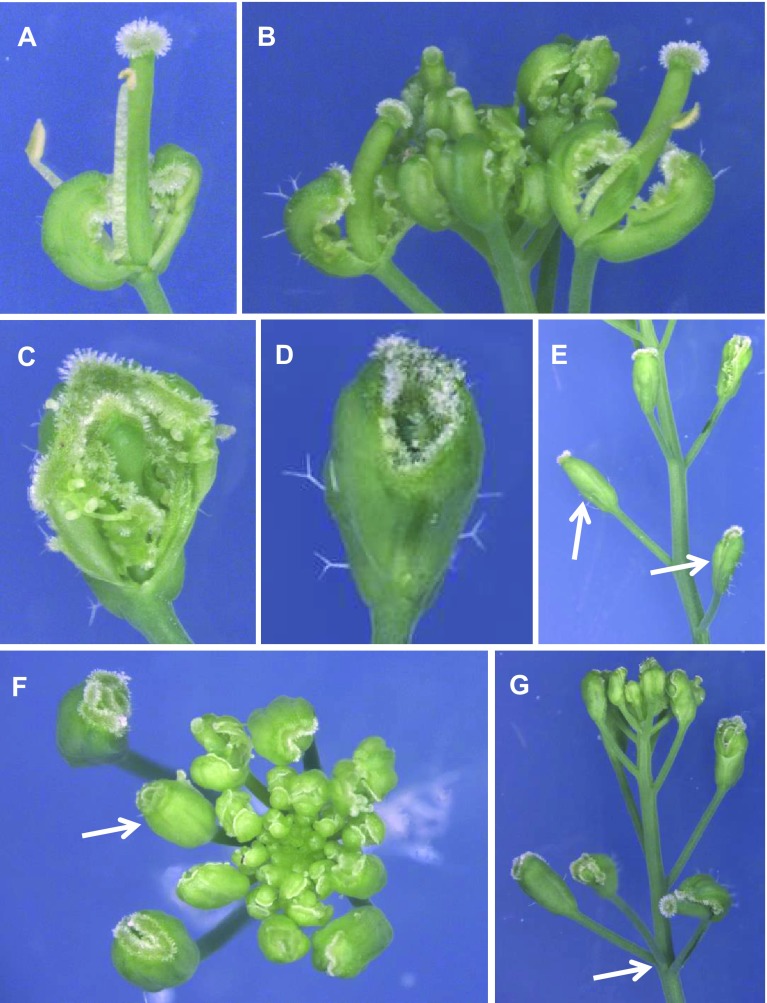
*erf12* enhances the *ap2* phenotype. An exemplary *ap2* flower (**a**) and inflorescence (**b**). Flowers of *ap2 erf12* plants (**c**, **d**). Inflorescences of *ap2 erf12* plants (**e**–**g**). Note the complete fusion of outer whorl organs (arrows) in (**e**) and the aberrant phyllotaxy (arrow) in (**g**)

Because *APETALA2* also functions in the floral transition and floral organ specification, we analysed the genetic interaction between *ap2*-*7* and *erf12* mutants by creating a double *ap2*-*7 erf12* mutant. *ap2*-*7* mutant flowers consisted of carpelloid sepals and bract-like structures, some stamens and a relatively normal gynoecium (Fig. 8a, b). However, *erf12* enhanced the *ap2* mutant phenotype: stamens were absent in *ap2 erf12* double mutant flowers and whorl one organs had a stronger carpeloid identity than in *ap2* single mutants (Fig. 8c, d) and were often completely fused and resembled a carpel (Fig. 8e, f), with trichomes. Similar to *erf12* single mutants, the floral phyllotaxy of *ap2 erf12* inflorescences was also defective (Fig. 8g), but was not more severe than that of single *erf12* mutants.

### Mutation of *ERF9* does not affect floral development

To characterise potential genetic redundancy between *ERF12* and other related AP2/clade VIIIa members, we analysed the phenotype of a T-DNA insertion mutant of ERF9 (Fig. 2c). *erf9* showed wild type floral and inflorescence development (data not shown) and when combined with *erf12*, no additional or enhanced phenotypic defects were observed in *erf9 erf12* plants to those of *erf12* single mutants.

## Discussion

### ERF12 and MFS1 are an angiosperm-specific invention

The MFS1 AP2 protein in rice, which determines floral meristem and organ identity, prompted a phylogenetic analysis, which identified the *Arabidopsis* orthologue as ERF12. The absence of ERF12/MFS1 homologues in plant radiations prior to the emergence of angiosperms and in *Amborella trichopoda*, the monotypic sister genus to all angiosperms, suggests that ERF12-type proteins represent a derived angiosperm trait subsequent to the divergence of eudicots from basal angiosperms that co-evolved with the diversification of angiosperm floral morphology. This contrasts with close AP2/ERF relatives belonging to clade VIIIb, which are more ancestral (Chandler 2018). This conclusion is supported by several other pieces of evidence. Firstly, no ERF12/MFS1 orthologue is present in the three gymnosperm genomes compared, nor in that of the magnoliid *Cinnamomum kanehirae*, another basal angiosperm, whose closest protein relatives group in the ERF3/7 clade. The robust and comprehensive analysis of plant taxa across plant kingdom (Fig. 1) show that an ERF12/MFS1 homologue is clearly shared by all early non-grass monocots analysed (*Ananas comosum*, *Musa acuminata*, *Phalaenopsis equisetum*, *Spirodela polyrhiza*, *Zostera marina*), and in all grass monocots and evolutionarily young eudicots analysed. Furthermore, the closest *Amborella* relative does contain an ESR domain, confirming that it belongs to the clade VIIIb proteins (Chandler 2018). Another argument is that ERF12 predominantly affects sepals, which are a relatively recent morphological innovation, since they are absent in *Amborella*, the best extant reference for understanding the evolutionary molecular genetic basis of flower development (Amborella Genome Project 2013) and also the magnoliid *Cinnamomum kanehirae*. The tepals of *Amborella* flowers show a gradual morphological transition from outer to inner floral organs and floral trait modelling suggests that flowers of the most recent angiosperm ancestor had an undifferentiated perianth consisting of multiple tepals (Sauquet et al. 2017). *Phalaenopsis equestris* also possesses tepals, but contains a protein that more closely aligns with ERF12 than other group VIIIa proteins (Fig. 1). This might represent an intermediate ancestor in the evolution of an ERF12 protein associated with sepal development and flowers having a characteristic angiosperm floral body plan. Widespread whole-genome duplication across angiosperms, leading to a diversification in gene family content, particularly in crucifers (Amborella Genome Project 2013), probably led to the coopting and neofunctionalisation of *ERF* genes such as *ERF12* for specific floral functions. During angiosperm evolution, perianth differentiation is thought to have multiple independent origins and many theories exist concerning the gene neo- and subfunctionalisations that underlay these events (Irish 2009). One example is the conversion of tepals into sepals into *Phalaenopsis equestris* brought about by silencing *SEPALLATA3* (Pan et al. 2014). However, ERF12 function is not associated with perianth organ identity, but organ merosity, and the phylogenetic analysis here that exploits the currently available sequenced genomes representing key plant evolutionary stages, robustly supports the hypothesis that ERF12 neofunctionalised comcomitant with the appearance of angiosperm flowers with a differentiated perianth.

In *Arabidopsis*, clade VIIIa AP2/ERF members except ERF8 and ERF12, phylogenetically associate as sister pairs or paralogues (ERF3 and ERF7; ERF4 and ERF9; ERF10 and ERF11). The absence of redundant paralogues might explain why single *erf12* mutants display a phenotype, whereas redundancy between ERF9 and ERF4 might mask *erf9* phenotypes. Few clade VIIIa ERF proteins have been functionally characterised, but ERF11, which is encoded by the adjacent locus to *ERF12*, promotes internode elongation via gibberellin synthesis (Zhou et al. 2016) and represses ethylene synthesis (Li et al. 2011). *ERF9* is involved in plant defence against necrotic fungi via ethylene/JA pathways (Maruyama et al. 2013) and ERF8 is a transcriptional repressor that negatively regulates ABA-mediated responses and is involved in immune signalling (Cao et al. 2018) and water stress in kiwifruit (Zhang et al. 2017). Although *ERF12* has been functionally implicated in diverse hormone pathways, such as auxin (Lewis et al. 2013), gibberellin (Cao et al. 2006), salicylic acid and methyl jasmonate (Caarls et al. 2017), the data here implicate it as the first ERF VIIIa clade member with a developmental role.

### Stereotypy in wild type floral organ number is initially unstable

Stochastic variation in floral organ number exists within populations or individuals of many species. For example, petal number in *Cardamine hirsuta* (Pieper et al. 2016) is naturally variable and together with stamen number, is regulated by seasonal temperature (Matsuhashi et al. 2012; McKim et al. 2017) and tepal number varies among the Ranunculaceae (Kitazawa and Fujimoto 2014). Stamens are numerically the most variable *Arabidopsis* organ, due to the frequent absence of lateral stamens (Anderson and Roberts 1998). In addition to an environmental and quantitative genetic basis for organ number variation, we show here that the number of wild type floral organs, especially of stamens, is initially developmentally labile and stabilises after the first few flowers. The floral transition thus involves variability in organ merosity, as well as meristem identity changes. The timing of stamen founder cell specification is unknown; auxin response maxima have been spatially associated with early developmental time points, but the founder-cell marker *DRNL* is expressed prior to *DR5* (Chandler et al. 2011) and non-robust positional information might result from the imprecise resolution of initially diffuse expression domains of positional regulators. Floral organ number and increased organ fusion and homeosis in wild-type *Arabidopsis* are enhanced by GA treatment (Plackett et al. 2018) and the most common homeotic conversions are petalloid stamens (Chandler and Werr 2011). Variable organ numbers in first-formed wild type flowers probably represents variable molecular stochasticity in expression levels or boundaries of homeotic or other regulatory genes (Kitazawa and Fujimoto 2014), especially considering the close proximity of petal and lateral stamen founder cell populations (Chandler et al. 2011).

### *ERF12* promotes the floral transition and floral meristem identity

The late-flowering phenotype of *erf12* mutants in LD and SD establishes that *ERF12* promotes the IM-to-FM transition independently of photoperiod. This contrasts with mutation of several *Arabidopsis* AP2-type genes that repress the floral transition, including *AP2* (Ohto et al. 2005), *SCHLAFMÜTZE* (Mathieu et al. 2009), *TARGET OF EAT1* (*TOE1*) and *TOE2* (Aukerman and Sakai 2003), and *GLOSSY15* in maize (Lauter et al. 2005). More cauline leaves in *erf12* than wild type in both photoperiods represents an extension of the meristem maturity phase, where phytomers transition from containing a secondary inflorescence meristem in the axil of a cauline leaf, to a floral meristem in the axil of a cryptic bract (Park et al. 2014). Despite repressing the floral transition, other *AP2*-type genes promote FM identity and simultaneously repress vegetative characteristics from flowers. Thus, sepals of *Arabidopsis ap2* mutants and *lip1 lip2* double mutants in *Antirrhinum* are often converted to leaf- or bract-like structures (Bowman et al. 1989; Keck et al. 2003) and *supernumerary bracts* in rice and double *indeterminate spikelet 1/sister of indeterminate spikelet 1* in maize display bracts instead of flowers (Lee et al. 2007; Chuck et al. 2008). Additionally, mutation of the *Arabidopsis* AP2/ERF gene *PUCHI* results in partial bract outgrowth (Karim et al. 2009), and of its orthologues *FRIZZY PANICLE* in rice (Komatsu et al. 2003) and *BRANCHED SILKLESS* in maize (Colombo et al. 1998), blocks the transition from spikelet to floral meristem identity. These mutant phenotypes can be interpreted as heterochronic effects, and the delayed or abolished transition from spikelet meristem or IM to FM produces ectopic bracts or leaf-like organs. The concept of the floral transition as a multidimensional process involving different spatiotemporal components is underlined by uncoupling of the floral transition and bolting by non-permissive conditions (Pouteau and Albertini 2011), which also causes the floral reversion of incompletely committed meristems in many species (Asbe et al. 2015). In *Petunia*, floral reversion has revealed a continuum of variation at the levels of meristem identity, primordium initiation and floral organ identity (Pouteau et al. 1998). Similarly, *Arabidopsis puchi* flowers partially spontaneously revert (Karim et al. 2009). Flower development is also a multistep process that invokes dynamic and competing ab-/adaxial polarity or centroradial models (Chandler and Werr 2014). Based on transcriptome analysis in pea where the *ERF12* homologue is expressed in the vegetative SAM (Wong et al. 2008), by analogy, *ERF12* might be present in the vegetative Arabidopsis meristem, raising questions whether it is upregulated in the IM either due to the endogenous age-related developmental programme or on the floral transition, and if this were the case, threshold models for its function could be invoked, or its functional specificity might depend on appropriately expressed interaction partners.

Sepal initiation occurs independently of a stem cell population as marked by *CLAVATA3* (*CLV3*) and *WUSCHEL* (*WUS*), which are expressed in IM stem-cells and in the FM centre from late floral stage 2, but are absent during early FM development (Goldshmidt et al. 2008; Yadav et al. 2009). We speculate that in addition to a delayed floral transition, *erf12* mutants have an extended meristem maturation phase in which FM identity and autonomy are irreversibly acquired, and thus a prolonged sepal initiation phase prior to the centroradial initiation of inner floral organs, leading to more sepals. Genes such as *AP1* and *AP2* that regulate FM identity in *Arabidopsis*, are also required for sepal identity (Litt 2007), presumably because FM identity is acquired during the sepal initiation phase, which involves unidirectional polarity (Chandler and Werr 2014). The stochastic variation in wild-type floral inner organs is enhanced in *erf12*, suggesting that *ERF12* also contributes to organ merosity during the centroradial phase of inner organ initiation. Although *erf12* shows photoperiod-independent late-flowering, the increased frequency of sepals, petals and lateral stamens in *erf12* is dependent on long-days. This might reflect the photoperiodic regulation of *ERF12* transcription (Mantegazza et al. 2014), or be because short-day flowering as a default pathway, is potentially more robust than the long-day promotion of flowering, which involves the activation and more precise convergence of pathways at a temporally specific point and might be thus more inherently unstable and prone to disruption.

Phyllotaxis at the apical meristem is largely regulated by auxin response maxima (Reinhardt et al. 2000); however, disrupted phyllotaxy can also result from meristematic effects that cause delayed floral primordium outgrowth, as in *arabidopsis histidine phosphotransfer protein 6* mutants (Besnard et al. 2014), or post-meristematic mechanisms that perturb internode elongation due to the inappropriate regulation of *CUP*-*SHAPED COTYLEDON* (*CUC*) 2 by miRNA164 (Peaucelle et al. 2007), as observed in higher-order *cuc* mutants (Burian et al. 2015). The basis of clustered siliques and disrupted phyllotaxis in *erf12* inflorescences is unknown, but appears at least partly to result from aberrant internode elongation.

### Aspects of the *erf12* mutant phenotype are complemented by *ERF12*

Supernumerary sepals in the first-formed flower was used as the most easily quantifiable phenotype to assess *erf12* complementation by the genomic *ERF12* locus. Transformation by *gERF12Up* was sufficient to complement the *erf12* sepal phenotype. This might reflect the higher sequence conservation revealed by phylogenetic shadowing in the upstream promoter region compared to downstream sequences. However, the late flowering phenotype of *erf12* was not complemented by the whole genomic sequence between the up- and downstream flanking genes, potentially reflecting the importance of the appropriate physical open chromatin context of *ERF12* within its native chromosomal environment, since epigenetic mechanisms are also major components of flowering time regulation (Ahmad et al. 2010).

### *ERF12* expression spatiotemporally coincides with the observed mutant phenotypes

Dynamic *pERF12::erGFP* expression in the IM and FMs throughout inflorescence development and not only in the first-formed flowers where the strongest floral mutant phenotypes were observed, suggests that these phenotypes also depend on the developmentally regulated expression of downstream targets. The ring of *pERF12::erGFP* expression at the base of young floral primordia as they emerge from the IM might mechanistically underlie a function for *ERF12* in the timing of primordium outgrowth and establishing phyllotaxy. Strong *pERF12::erGFP* expression in the abaxial side of floral stage 1 and 2 FMs, during the sepal initiation phase coincide with the *erf12* sepal phenotype and strong foci of *pERF12::erGFP* expression in the intersepal regions might be the basis for sepal fusion, and expression throughout the FM encompasses the sites of stamen and petal founder cells (Chandler et al. 2011). In summary, *pERF12* expression data spatiotemporally coincide with all aspects of the mutant phenotypes. Furthermore, *ERF12* expression in cotyledon and leaf guard cells shows that the gene is active in embryonic and postembryonic tissue in differentiated cell types as well as in meristematic tissue.

### ERF12 and MFS1 are functionally similar

A comparison between the functions of *ERF12* and rice *MFS1* must consider the different floral morphologies between both species. Wild-type rice spikelets contain two rudimentary glumes, two sterile lemmas, in addition to a terminal floret with a single lemma and palea in whorl 1, two lodicules in whorl 2, six stamens in whorl 3, and one carpel in whorl 4. The lodicules are considered by some to be modified petals (Whipple et al. 2007). The lemma and palea have historically been interpreted as extra-floral organs, as a bract and prophyll, respectively (Lombardo and Yoshida 2015). However, because they express floral genes and based on rice floral homeotic mutants, they are probably sepal analogues (reviewed in Lombardo and Yoshida 2015), with the palea potentially representing a differentiated lemma (Ambrose et al. 2000). In *mfs1* mutants, the sterile lemma is homeotically converted into the rudimentary glume and the majority of *mfs1* spikelets contain an extra lemma-like organ, often two degenerated palea-like organs instead of one, and a variable number of stamens (Ren et al. 2013). Furthermore, an enlarged FM suggests that spikelets with an extra lemma might derive from two florets, due to a delay in FM determinacy acquisition. Thus, the pleiotropic *mfs1* phenotype consists of three components: a delayed spikelet meristem to FM transition, organ homeoses (sterile lemma/glume and palea) and increased organ merosity (supernumerary lemmas and paleae). Despite the absence of intermediate meristems in *Arabidopsis* equivalent to the rice spikelet meristem, delayed flowering of *erf12* mutants represents a similar delay in establishing FM identity to *mfs1*. No organ homeoses were observed in *erf12*, but this aspect of the *mfs1* phenotype is probably masked, because in contrast to lemma and palea, *Arabidopsis* sepals are morphologically similar and no analogues of sterile lemmae or rudimentary glumes are present. Conversely, rice has no analogous organs to petals, but the variable number of stamens and sepals in *erf12* phenocopies labile numbers of stamens, lemmas and paleae in *mfs1*. The largely congruent *erf12* and *mfs1* mutant phenotypes suggests that MFS1 and ERF12 are functionally equivalent, which is further supported by the high conservation of the AP2 domain and the shared C-terminal EAR-like motif. The *erf12* phenotype manifests within a narrow developmental time-window including the first-formed flowers, which suggests a heterochronic function for *ERF12*; but no evidence is available concerning changes in the severity of the *mfs1* phenotype along the rachis, the timing of the floral transition or defects in phyllotaxy.

### *ERF12* interacts genetically with closely related *ERF* factors and *AP2*

Genetic interactions between *ERF12* and its closely related *ERF* clade VIIIb genes *DRN*, *DRNL* and *PUCHI* were revealed by higher-order mutant analysis. Loss of *ERF12* function in the *drn drnl puchi* triple mutant background led to attenuated phenotypes, suggesting that the phenotypic severity of *drn drnl puchi* plants requires *ERF12* function. Conversely, the supernumerary sepal phenotype of *erf12* single mutants was counteracted by loss of *DRN*, *DRNL* and *PUCHI* function. Thus, *ERF12* and the combined functions of *DRN*, *DRNL* and *PUCHI* are opposing in terms of the repression and promotion of organ initiation, respectively. DRN, DRNL and PUCHI are considered to be transcriptional activators (see Chandler 2018) and all contain a transcriptionally active ESR domain (Nomura et al. 2009). Conversely, ERF12 contains a C-terminal EAR-like repressor domain, which might transcriptionally repress downstream targets at the timepoint of incipient FM development within the IM, which is associated with large-scale gene downregulation (Wellmer et al. 2006). ERF12 also contains an AP2 transcriptional activation domain that might facilitate protein–protein interactions (Chandler et al. 2007); thus, ERF12 might activate and repress distinct subsets of downstream targets, similar, to AP2 (Yant et al. 2010). The genetic interactions between *ERF12* and *DRN*, *DRNL* and *PUCHI* might represent the combined outread of cumulative activated and repressed targets. Although scant detailed knowledge concerning direct gene targets for these genes currently limits further interpretation of the interactions, it demonstrates that all genes converge on similar downstream targets. In rice, *MFS1* transcriptionally activates the *AP2* homologues *SNB* and *OsIDS1* (Ren et al. 2013). Here, the *ap2* phenotype is dependent on *ERF12* and is enhanced by *erf12*. In strong *ap2* alleles, sepals are converted into carpels, petals are absent and the number of stamens is reduced, whereas *erf12 ap2* mutants only produce carpels. This synergistic phenotype is similar to that of *ap2 ant* double mutants, which only produce carpels (Elliott et al. 1996). This potentially reflects the regulation of *AP2* transcription or its expression domain by ERF12. AP2 possesses a cadastral role in regulating *AGAMOUS* expression (Drews et al. 1991) and potentially loss of *AP2* and *ERF12* function regulate downstream B-function genes to abolish stamens.

The absence of observable mutant phenotypes for *erf9* or genetic interactions in combination with *erf12* suggests that either it is not involved in development, or functions redundantly with other related *ERF* genes, which requires confirmation via a more systematic higher order mutant analysis of group VIIIa members.

**Fig. 9:**
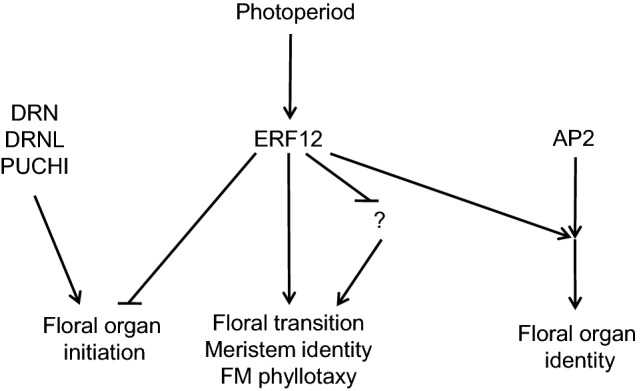
Model for the integration of ERF12 function into other genetic pathways and developmental processes. *ERF12* function is dependent on photoperiod and contributes pleiotropic roles to floral organ initiation and floral meristem identity. ERF12 is a putative transcriptional repressor and opposes the functions of the related AP2 transcription factors DORNRÖSCHEN (DRN), DORNRÖSCHEN-LIKE (DRNL) and PUCHI, and integrates into the APETAL2 (AP2) pathway to regulate floral organ identity. Alternatively, ERF12 might repress unknown growth or floral transition activators, leading to late flowering and organ overproliferation when this repressive function is lost in the mutant

In summary, we have characterised pleiotropic roles of the ERF12 transcription factor in reproductive development (Fig. 9): firstly, it promotes the floral transition and specifies FM identity; secondly, dependent on long-day photoperiods, it represses floral organ initiation, particularly of sepals and stamens; supernumerary *erf12* sepals potentially arise from an extension of the FM maturity phase. Thirdly, as a putative repressor, ERF12 counteracts the promotion of floral organ initiation by the combined activities of the related transcription factors DRN, DRNL and PUCHI and stabilises the inherent lability of organ number of the first-formed wild type flowers. Fourthly, ERF12 converges with the AP2 pathway to specify organ identity. Finally, ERF12 is continuously required in the IM to maintain appropriate FM phyllotaxy. Alternatively, we can also speculate that the observed *erf12* phenotypes could result from the loss of a wild-type function that represses activators of the floral transition or growth, such that in the mutant, later flowering and overgrowth, including supernumerary organs arising under certain physiological conditions. This study is confined to a single mutant allele (also for *erf9*), because no others are currently available in public stock centres but the caveat exists that we cannot completely exclude that these alleles are null, especially *erf12*, because the T-DNA insertion is in the 3′ region of the open reading frame. However, the successful complementation and conserved functions with rice MFS1 convincing and unequivocally consolidate the interpretations and conclusions concerning ERF12 functions in floral organogenesis. The discovery of a novel gene function that contributes to phase change and floral organogenesis suggests that the genetic network involved in these processes might not yet be fully elucidated, even at a non-redundant genetic level. ERF12 also enhances our understanding concerning the incremental processes that comprise the floral transition, and the stochastic instability in wild-type floral organ merosity reveals that the floral transition also focuses positional information required to appropriately generate floral organ founder cells in the FM, in addition to establishing meristem identity.

## Electronic supplementary material

Below is the link to the electronic supplementary material.

Supplementary material 1 (DOCX 14 kb)
