# Supplementary material for: A phylogenetically conserved APETALA2/ETHYLENE RESPONSE FACTOR, ERF12, regulates Arabidopsis floral development
Source: Plant Mol Biol. 2019 Dec 5;102(1):39–54. doi: 10.1007/s11103-019-00936-5 (PMC6976583; doi:10.1007/s11103-019-00936-5)
Supplement: Supplementary file 1 — Supplementary material 1 (DOCX 14 kb) [file 11103_2019_936_MOESM1_ESM.docx]

**Table S1. Primers used in this research**

ERF12F 5´-AATACAATTTATTTACAAACTC-3´

ERF12R 5´-CTCTAGATTCTCTCATATTTC-3´

LB3 5´-TAGCATCTGAATTTCATAACCAATCTCGATACA-3´

ERF9F 5´-ATGGCTCCAAGACAGGCGAAC-3´

ERF9R 5´-CTAAACGTCCACCACCGGTGG-3´

LBa1 5´-TGGTTCACGTAGTGGGCCATCG-3´

promERF12F 5´-GGCGCGCCAGTTGCAGAAGCAAAGAGAGT-3´

promERF12R 5´-CCCGGGTGTTAGACTCTCAAGAGGGGA-3´

gERF12UpR 5´-GGCGCGCCAATACAATTTATTTACAAACTC-3´

ERF12UpRnew 5´-CCCGGGAATACAATTTATTTACAAACTC-3´

gERF12DSF 5´-CCCGGGTTTTATTTATTTTGAAAATTTTC-3´

gERF12DSR 5´-CCCGGGCGCGCCAGATTTAATTATGATTATTAG-3´

pGPTVF 5´-ACCGCCCAGTCTAGCTATCGC-3´

pGPTVR 5´-GTTGGGGTTTCTACAGGACGTAACAT-3´

ERF12Fgeno 5´-TAAGATCAACAACCTTATATG-3´

ERF12Rgeno 5´-TTACTAGTCAGTGTGATTCAC-3´
